# Hydration Efficacy of a Milk Permeate-Based Oral Hydration Solution

**DOI:** 10.3390/nu12051502

**Published:** 2020-05-21

**Authors:** Craig W. Berry, S. Tony Wolf, Bob Murray, W. Larry Kenney

**Affiliations:** 1 Department of Kinesiology, The Pennsylvania State University, University Park, State College, PA 16802, USA; saw85@psu.edu (S.T.W.); w7k@psu.edu (W.L.K.); 2 Sports Science Insights, LLC, Crystal Lake, McHenry County, IL 60014, USA; bob@sportsscienceinsights.com; 3 Graduate Program in Physiology, The Pennsylvania State University, University Park, State College, PA 16802, USA

**Keywords:** dairy, sports drink, beverage hydration index, osmolality, electrolytes, fluid balance, plasma volume

## Abstract

Milk permeate is an electrolyte-rich, protein- and fat-free liquid with a similar carbohydrate and mineral content to that of milk. Its hydration efficacy has not been examined. The beverage hydration index (BHI) has been used to compare various beverages to water in terms of post-ingestion fluid balance and retention. Our purpose was to compare the BHI (and related physiological responses) of a novel milk permeate solution (MPS) to that of water and a traditional carbohydrate–electrolyte solution (CES). Over three visits, 12 young subjects consumed 1 L of water, CES, or MPS. Urine samples were collected immediately post-ingestion and at 60, 120, 180, and 240 min. BHI was calculated by dividing cumulative urine output after water consumption by cumulative urine output for each test beverage at each time point. The BHI for MPS was significantly higher at all time points compared to water (all *p* < 0.001) and CES (all *p* ≤ 0.01) but did not differ between CES and water at any time point. Drinking 1 L of MPS resulted in decreased cumulative urine output across the subsequent 4 h compared to water and CES, suggesting that a beverage containing milk permeate is superior to water and a traditional CES at sustaining positive fluid balance post-ingestion.

## 1. Introduction

Maintaining adequate hydration, in both unchallenged and dehydrated conditions, is associated with multiple health benefits. Proper hydration reduces risk for the development of chronic diseases, including cardiovascular, metabolic, and renal diseases, including the development of kidney stones [1]. Additionally, adequate hydration is associated with reductions in cognitive [2,3,4] and athletic performance [5,6] impairments. Carbohydrate (CHO)–electrolyte solutions (CES), including sports drinks and oral rehydration solutions have traditionally been the options of choice for promoting euhydration [7,8,9]. These solutions are designed to maintain or improve hydration status by promoting drinking, absorption of fluid from the small intestine via activation of sodium-glucose transporters [10], and retention of fluid within the body and the vascular compartment [11,12]. These pro-hydration properties are primarily a function of the carbohydrate and electrolyte composition of the beverage, as well as its total osmolality [13,14,15,16,17].

Maughan et al. [14] proposed the beverage hydration index (BHI) in 2016 as a measure of the hydrating capacity or efficacy of a given beverage relative to water. Since that time, BHI has been used to compare the hydration efficacy of a variety of beverages [14,18,19]. This index is able to assess how a beverage impacts post-ingestion body fluid balance in individuals independent of sex or body mass [19]. In calculating BHI, cumulative urine output at various time points following consumption of 1 L of water is set to a value of 1.0. Following beverage consumption of the same volume as water, beverages that elicit a greater urinary excretion than water over a fixed period have a BHI less than 1.0, while those that elicit greater fluid retention and attenuated urinary excretion have a BHI greater than 1.0. This measurement thereby enables comparisons of the hydration capacity of various beverages both within and across studies. Although the 2-h post-ingestion time point was proposed by Maughan as the standard for comparison [14], additional information can be gleaned from data throughout the entire 4-h testing period, especially for older adults [18,20] for whom ingested beverages are retained for a longer period of time, or after consumption of beverages that maintain positive fluid balance beyond 2 h.

Dairy-based beverages have been suggested as efficacious alternatives to traditional sports drinks [21]. Maughan et al. determined that the BHI was higher for both whole milk and skim milk compared to water, and similar to that of an oral rehydration solution (ORS) [14]. Those investigators opined that the high BHI of milk was likely due to its high protein (and perhaps fat) content, while the elevated BHI of the ORS was due to its carbohydrate and electrolyte content. To harness the hydrating qualities of dairy, attempts have been made to develop hydration beverages from byproducts produced during ultrafiltration of milk and cheese products [22,23,24,25]. For example, large quantities of milk permeate are produced as a byproduct of the ultrafiltration of milk. Milk permeate is a protein-free, fat-free liquid that contains the approximate carbohydrate and mineral content of milk [24]. It is also high in sodium and potassium and has a relatively high osmolality (primarily due to its total mineral content). Therefore, a solution containing milk permeate, developed primarily for use during exercise and other dehydrating conditions, may have hydration characteristics that are, at a minimum, similar to that of a traditional CES beverage. However, it remains to be determined how a milk permeate-based solution (MPS) impacts hydration status in humans and how this hydration efficacy compares to that of other commercial hydration solutions.

Determining the BHI of various beverages is an important first step in determining hydration efficacy, since the conditions under which this index is calculated are highly standardized and well-described [14], and because it has been measured in individuals varying in size, sex, and age [14,18,19]. Therefore, the primary purposes of the present study were: (1) to determine the hydration efficacy of a novel beverage containing milk permeate relative to water and CES, as measured by net fluid balance and BHI; and (2) to determine the extent to which fluid and electrolytes are retained in the vascular space after ingestion of each solution. We hypothesized that both CES and MPS would demonstrate a higher BHI than water over a 4-h period after standardized beverage ingestion in euhydrated subjects and that the BHI of the MPS beverage would be similar to that of the CES beverage.

## 2. Materials and Methods

### 2.1. Study Population

Twelve young men and women (23 ± 1 years) participated in the study. Subjects were recruited from the community in Centre County, PA using advertisements or from a pool of individuals who had participated in previous studies. All subjects underwent a screening visit consisting of anthropomorphic measurements, resting heart rate and blood pressure, and blood chemistry prior to enrollment. Subjects were excluded if they had reported any prior history of renal, metabolic, prostate, or cardiovascular disease or if they were taking any medications that may impact fluid balance. All procedures were approved in advance by the Pennsylvania State University Institutional Review Board, and all subjects gave written or verbal consent before participation in accordance with the Declaration of Helsinki. All testing was conducted in Noll Laboratory at the Pennsylvania State University.

### 2.2. Study Design

Subject characteristics are displayed in Table 1. Twelve young men and women participated in the study. Ten subjects completed all three trials, one subject completed the water and MPS trials only, and one subject completed the CES and MPS trials only (due to COVID considerations). Statistically valid mean substitutions were used for the two missing trials during statistical analysis [26]. Subjects completed trials in a randomized order. All trials began between 0600 and 0900.

The study design followed that of Maughan et al. [14], further described by Clarke et al. in our lab [18]. Prior to each experimental trial, subjects fasted overnight for at least 8 h, refrained from alcohol and caffeine consumption for 12 h, and refrained from vigorous physical activity for 24 h. Additionally, subjects were not permitted to ingest any food for the duration of each trial. This total period of food restriction did not present any adverse consequences to any subjects. Subjects were instructed to maintain normal fluid intake in the 24 h prior to each study. One hour before arriving at the laboratory, subjects consumed 500 mL of spring water (Aquafina, PepsiCo, Harrison, NY, USA). Subjects self-reported that they had consumed 500 mL of spring water. Upon arrival to the laboratory, subjects entered a thermoneutral room (16–20 °C, 20–30% relative humidity), where they remained seated in a semi-recumbent position for the duration of the study, except during urine collection. Subjects voided their bladder in a 1-L plastic urine container (designated as the “pre” time point for data presentation) upon arrival to the laboratory. Pre-trial hydration status was assessed using a urine refractometer to determine urine specific gravity, with values between 1.000 and 1.025 confirming euhydration. Following voiding of the bladder, each subject’s near-nude body mass was measured. Subjects then sat for 10 min before an intravenous catheter was inserted into an antecubital vein. A 10-mL pre-ingestion venous blood sample (“pre” time point for blood) was then collected.

**Figure 1:**
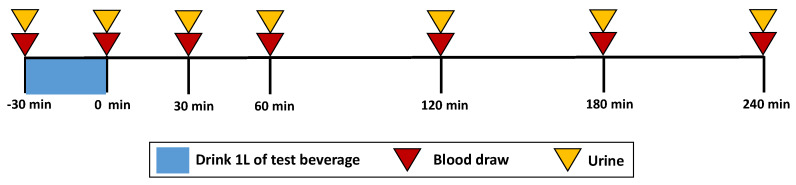
Timeline of measurements. Blood samples were collected immediately before (−30 min) and after (0 min) consuming the final beverage aliquot and at 30, 60, 120, 180, and 240 min post-ingestion. Urine samples were collected following each blood sample except at the 30 min time point, i.e., at 0, 60, 120, 180, and 240 min post-ingestion.

Following collection of the “pre” time point blood sample, subjects ingested 1-L of a randomly assigned test beverage in 4 equal aliquots over a 30 min period (0.25 L every 7.5 min). Blood samples were collected immediately after completing the final aliquot (0 min) and at 30, 60, 120, 180, and 240 min post-ingestion. Blood samples were collected in 2 serum separator tubes (4 mL each) to measure serum electrolytes and serum osmolality and 1 K_2_ EDTA tube (2 mL) to measure hematocrit and hemoglobin. Urine samples were collected in 1-L urine containers following each blood sample, i.e., at 0, 60, 120, 180, and 240 min post-ingestion. At each time point, subjects were instructed to completely empty their bladder to the extent possible. If a subject needed to void their bladder between collection points, urine was collected and added to the urine sample of the following designated time point. A protocol timeline is outlined in Figure 1.

### 2.3. Test Beverages

Subjects completed the protocol three times, once for each test beverage. Trials were completed in a randomized order (random number generator) and separated by at least one week. The test beverages were distilled water, CES, and MPS. Beverage composition is displayed in Table 2. Beverage osmolality was tested in triplicate using a freezing-point osmometer (Model 3320, Advanced Instruments, Inc., Norwood, MA, USA); other reported values are label values. All beverage containers were kept sealed and stored at 16–20 °C prior to consumption.

### 2.4. Urine and Serum Analysis

Urine mass was assessed using an electronic balance accurate to the nearest 0.1 g, with the mass of the empty 1-L container subtracted from the weighed value. Serum separator tubes were left in an upright position for 30 min to allow serum clotting to occur. Following this 30-min period, blood samples were centrifuged (10 min, 4 °C, 4000 rpm).

Urine sodium and potassium concentrations at each time point were measured in triplicate (SmartLyte, Diamond Diagnostics). Urine and serum osmolality were measured at each time point in triplicate using a freezing-point osmometer (Model 3320, Advanced Instruments, Inc.). These analyses were conducted in our laboratory the same day of sample collection. Hematocrit, hemoglobin concentrations, glucose concentrations, serum electrolyte concentrations, and creatinine concentrations were also analyzed for each time point (Quest Diagnostics) within two days of sample collection for each trial.

### 2.5. Data and Statistical Analysis

Baseline blood chemistry and urine analysis were compared among drinks by repeated measures ANOVA to confirm that baseline hydration and renal function status were similar across trials. Main outcomes for this study were cumulative urine output (utilized to calculate BHI and net fluid balance), changes in plasma volume, and plasma glucose responses. BHI was calculated as the cumulative urine output of water divided by the cumulative urine output of the other two beverages at each time point. Plasma volume changes were calculated from hematocrit and hemoglobin concentration using the method of Dill and Costill [27]. Net fluid balance was calculated by subtracting the cumulative urine output at each time from the 1000 g of fluid consumed at the beginning of each trial. Subjects were in positive fluid balance if the obtained value was >0, and in negative fluid balance if this value was <0. Free water clearance (CH_2_O) was calculated as CH_2_O = V̇ – C_osm_, where *V̇* is urine flow in mL/min and C_osm_ is osmolar clearance (mL/min) = U_osm_ × V̇/P_osm_, with *U_osm_* and *P_osm_* being urine and plasma osmolality (mOsm/kg), respectively. Urine flow was calculated as urine output (mL) at each time point divided by the amount of time since last urine excretion (minutes).

Based on prior publications [14,18], an α < 0.05, and power = 0.8, we determined a priori that a minimum of 10 subjects would be needed to determine statistically significant differences among beverages. Data were analyzed in SAS (Cary, NC, USA) using PROC MIXED two-way ANOVA (beverage × time). With the exception of box plots, all data are presented as mean ± SD. Statistical significance was set a priori at α < 0.05.

## 3. Results

### 3.1. Cumulative Urine Output, Net Fluid Balance, and Beverage Hydration Index

**Figure 2:**
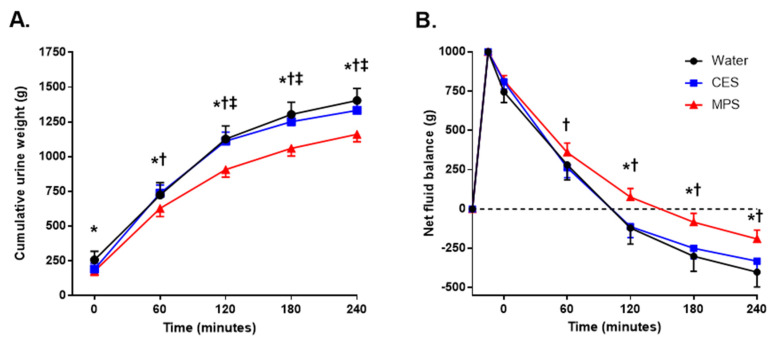
Cumulative urine output (**A**) and net fluid balance (**B**) 4 h after consumption of water, a carbohydrate–electrolyte solution (CES), and a milk permeate-based solution (MPS). Cumulative urine output was significantly lower in MPS compared to both water and CES at all time points after ingestion and was lower in CES compared to water beginning at 120 min. Net fluid balance was more positive for MPS compared to water and CES from 2 h onward. There were no differences in net fluid balance between the water and CES trials. Values are means ± SD. Differences among beverages were assessed by two-way ANOVA. * *p* < 0.05 MPS compared to water; † *p* < 0.05 MPS compared to CES; ‡ *p* < 0.05 CES compared to water.

Baseline (immediately before beverage ingestion) serum and urine markers of hydration and renal function status are displayed in Table 3. All measurements were similar across trials (all *p* ≥ 0.05). As shown in Figure 2A, cumulative urine output was significantly lower for MPS than for the water (*p* = 0.02) and CES trials beginning at 60 min (*p* = 0.01) and remained lower for the remainder of the trial (*p* < 0.01). Cumulative urine output was lower for the CES trial compared to water at 120, 180, and 240 min (*p* ≤ 0.03). The final (4-h) cumulative urine outputs for each trial were: water = 1423 ± 277 mL; CES = 1332 ± 234 mL; and MPS = 1191 ± 194 mL.

Figure 2B displays net fluid balance for each beverage. Net fluid balance was higher (more positive) for MPS compared to CES beginning at 60 min (*p* = 0.04) and remained so through the subsequent 3 h (*p* < 0.01) and higher than water from 120 to 240 min (*p* < 0.01). There were no differences in net fluid balance between the water and CES trials at any time point.

**Figure 3:**
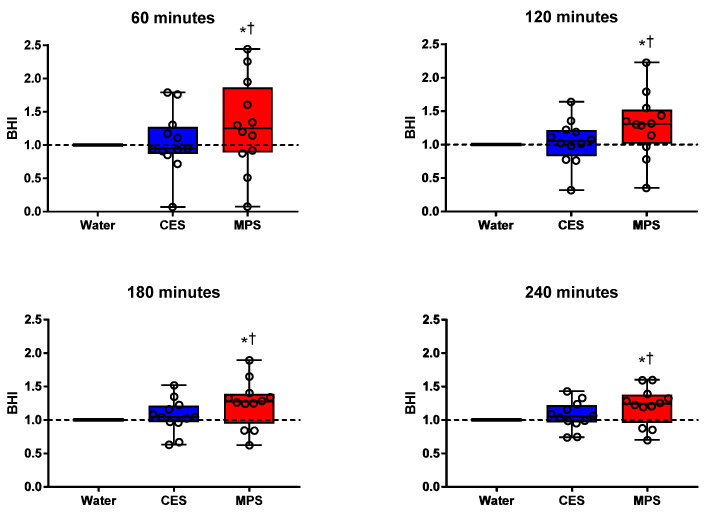
Beverage Hydration Index (BHI) for a carbohydrate–electrolyte solution (CES) and a milk permeate-based solution (MPS) relative to water (BHI = 1). The BHI for MPS was significantly higher compared to both water and CES at all time points after ingestion. BHI did not differ between water and CES at any time point. Boxes represent first and third quartiles with median values denoted by the horizontal line, while whiskers indicate minimum and maximum observations. Individual subjects’ BHIs are displayed as open circles. Differences between beverages were assessed by two-way ANOVA. * *p* < 0.05 MPS compared to water; † *p* < 0.05 MPS compared to CES.

**Figure 4:**
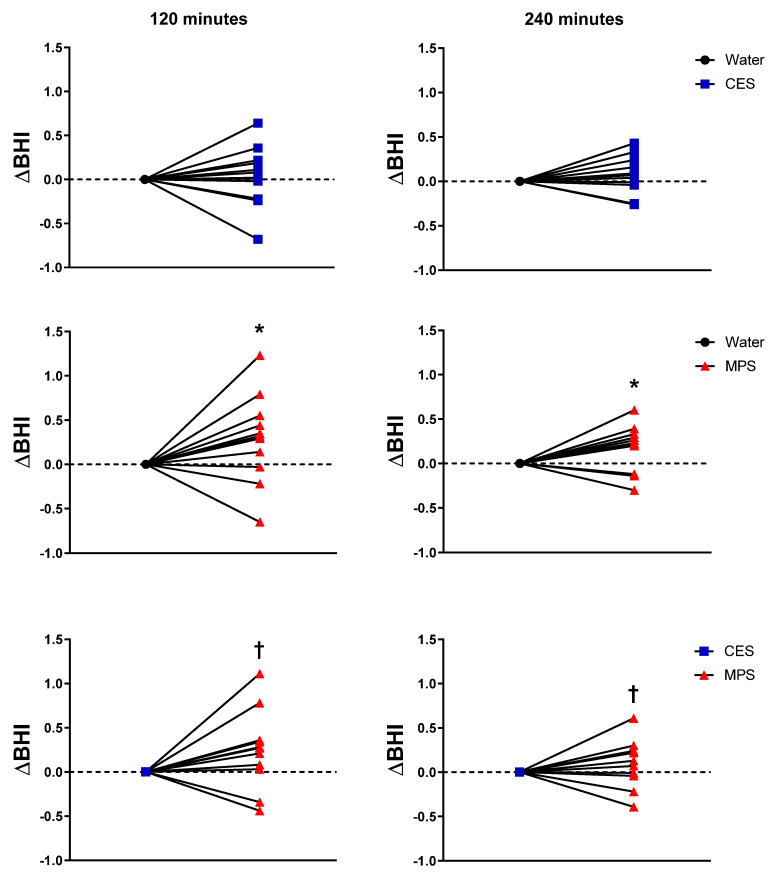
Data for individual subjects showing the difference in BHI for paired beverages. Differences between beverages were assessed by two-way ANOVA. * *p* < 0.05 MPS compared to water; † *p* < 0.05 MPS compared to CES.

Figure 3 presents box plots for BHI along with individual subject data. BHI was significantly higher for MPS than water and CES (all *p* ≤ 0.01) at all time points. BHI for CES did not differ statistically from water at any time point. Figure 4 displays the differences in BHI for each individual subject between pairs of beverages to better illustrate intra-subject differences (CES = 1.04 ± 0.09; MPS = 1.29 ± 0.15 at 120 min; CES = 1.07 ± 0.06 and MPS = 1.21 ± 0.09 at 240 min).

### 3.2. Serum and Urine Electrolyte Concentrations and Osmolalities

Serum and urine electrolyte concentrations, osmolalities, and free water clearance (CH_2_O) across time points are displayed in Table 4. There were no differences in serum or urine osmolality or electrolyte concentrations among trials prior to beverage ingestion (*p* ≥ 0.43). Immediately post-ingestion, serum osmolality declined in the water trial (*p* = 0.02) but increased in the MPS trial (*p* = 0.04). CES serum osmolality did not significantly change throughout the trial (*p* ≥ 0.23). Serum osmolality was greater in the MPS trial at 0, 30, and 60 min compared to water (all *p* < 0.001) and at 60 min compared to CES (*p* < 0.01).

Serum sodium decreased in all trials immediately post-ingestion (*p* ≤ 0.04). Serum sodium was elevated in MPS (*p* < 0.01) and CES (*p* < 0.01) compared to water at 30 min and higher for CES compared to both water (*p* = 0.04) and MPS (*p* < 0.01) at 60 min. There were no differences among beverages at 120 min or beyond (*p* ≥ 0.22). Serum potassium was significantly elevated at all time points in the MPS trial (all *p* < 0.001), but only at 120 min and beyond in the CES trial (*p* < 0.001). Serum potassium was significantly higher in the MPS trial compared to water at all time points (all *p* < 0.01), and at 0, 30, 60, and 120 min compared to CES (all *p* < 0.002).

Urine osmolality was higher in MPS compared to water at 120 min and beyond (all *p* < 0.01) and compared to CES at 60, 120, and 180 min (all *p* < 0.02). There were no differences in urine osmolality between water and CES at any time point. Urine Na^+^ concentration was significantly higher in MPS compared to water and CES at 120 and 180 min (*p* < 0.01) and urine K^+^ was higher in MPS compared to compared to water at 120 min and beyond (*p* < 0.01) and vs. CES at 120 min (*p* < 0.01). Free water clearance (CH_2_O) was lower in MPS compared to water through 180 min (all *p* < 0.02) and compared to CES through 120 min (all *p* < 0.04).

### 3.3. Plasma Volume

**Figure 5:**
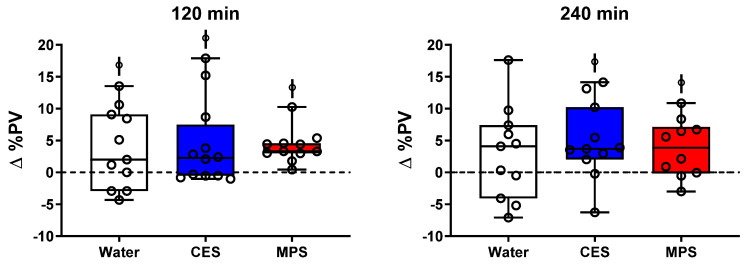
Changes in plasma volume at 120 and 240 min post-ingestion. Plasma volume was significantly elevated in all three beverages compared to Pre at 120 min and only in CES and MPS at 240 min. There were no significant differences in changes in plasma volumes between beverages at both time points. Boxes represent first and third quartiles with median values denoted by the horizontal line, while whiskers indicate minimum and maximum observations. Individual subjects’ changes in plasma volume are displayed as open circles. Differences between beverages were assessed by two-way ANOVA. ꬹ *p* < 0.05 compared to Pre.

There was a significant increase in plasma volume (∆PV) compared to pre-ingestion beginning at 30 min and continuing for the duration of the study for both MPS (3.5–4.0%; *p* < 0.03) and CES trials (3.8–6.7%; *p* < 0.02), but only at 120 min in the water trial (3.6%; *p* = 0.03). Changes in plasma volume are depicted at 120 and 240 min in Figure 5 as box plots with individual subject data shown. ∆PV was significantly higher for all three beverages compared to pre-ingestion at 120 min (all *p* < 0.04) but only for CES and MPS by 240 min (*p* < 0.02). All subjects experienced a positive change in plasma volume at 120 min in the MPS trial, with relatively small variation in the MPS trial, especially at 120 min.

### 3.4. Plasma Glucose Responses

**Figure 6:**
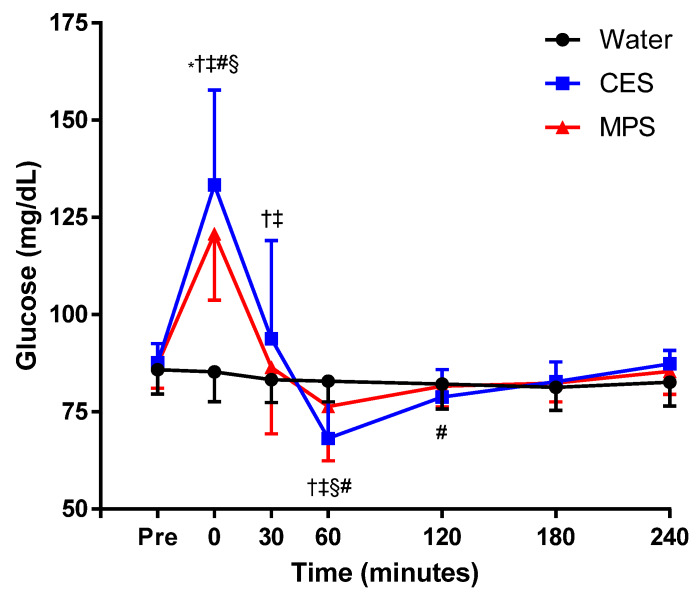
Plasma glucose concentration before and after consumption of water, a carbohydrate–electrolyte solution (CES), and a milk permeate-based solution (MPS). Glucose concentrations were significantly higher in CES and MPS compared to water immediately after and 30 min after ingestion of solutions. CES was higher than both MPS and water immediately after ingestion but lower at 60 min post-ingestion. Values are means ± SEM. Differences between beverages were assessed by two-way ANOVA. * *p* < 0.05 MPS compared to water; † *p* < 0.05 MPS compared to CES. ‡ *p* < 0.05 CES compared to water. § *p* < 0.05 MPS compared to pre. # *p* < 0.05 CES compared to pre. & *p* < 0.05 water compared to pre.

There were no differences in fasting plasma glucose concentration across trials prior to beverage ingestion (Figure 6). Immediately following ingestion (i.e., time 0) of the CES and MPS beverages (but not water; *p* = 0.86), there were significant increases in glucose concentration (both *p* < 0.01). There were no differences in plasma glucose concentration between water and MPS trials after 30 min, but plasma glucose concentration in the CES trial remained elevated compared to both water (*p* < 0.01) and MPS (*p* = 0.03) at 30 min and then lower than both water (*p* < 0.01) and MPS (*p* = 0.02) at 60 min. Plasma glucose concentration for all three beverages returned to pre-ingestion baseline values by 180 min (*p* ≥ 0.12).

## 4. Discussion

The present investigation examined the hydration efficacy of a novel milk permeate-based (MPS) solution in comparison to water and a traditional carbohydrate-based electrolyte sports drink (CES). Compared to CES, MPS had a lower carbohydrate content (4% vs. 6%), a similar sodium concentration (20 vs. 21 mmol/L), a higher potassium concentration (28 vs. 3.2 mmol/L), and higher osmolality (621 vs. 326 mOsm/L). The primary finding of the study was that 1 L of the milk permeate solution, consumed in a euhydrated state, was retained in the body longer compared to water and CES. Calculated BHI was significantly higher for MPS compared to both water and CES across the 4 h post-ingestion period, accompanied by a similar vascular fluid compartment expansion. Finally, there were blunted plasma glucose concentration excursions (i.e., immediate post-drinking increase and subsequent decrease below baseline at 1 h) following MPS ingestion compared to CES.

Maintaining proper hydration status at rest is important for its health benefits and in the prevention of developing chronic disease [1] and deficits in cognitive function [2,3,4] and athletic performance [5,6]. Carbohydrate-based electrolyte solutions have traditionally been recommended for promoting fluid retention and restoring euhydration [7,8,9], especially during and following physical activity that is accompanied by profuse sweating. The beverage hydration index (BHI), based on cumulative urine output and net fluid balance, is an innovative approach for assessing the hydration efficacy of different beverages [14,18,19]. This index is not impacted by differences in sex or body mass [19], allowing its application to the general population, including older adults [18]. In addition, results can be compared across studies that have followed the standardized BHI protocol published by Maughan et al. [14]. For example, following consumption of a traditional carbohydrate-based sports beverage, BHI values in our study were similar to those reported in young men and women by Clarke et al. using a similar CES beverage [18].

Prior investigations have suggested that whole or skim milk is an effective hydration solution at rest and its BHI is comparable to that of an oral rehydration solution beverage [14,28,29]. Milk contains electrolytes, proteins, minerals, and other solutes that, when absorbed in the small intestine, promote fluid retention. The milk permeate beverage contains the approximate carbohydrate and mineral content of milk [24], but without fats or proteins. The milk permeate solution tested here comprised approximately 21 mmol/L of sodium, which is similar to that of traditional carbohydrate-based hydration solutions, including the CES beverage utilized in this study (20 mmol/L). However, the potassium concentration of the MPS beverage (28 mmol/L) was considerably higher than the CES beverage (3.2 mmol/L). The MPS beverage also had a higher osmolality (621 ± 5 mosm/kg) compared to the CES beverage (326 ± 3 mosm/kg). The additional osmolar constituents of MPS consisted of chloride, magnesium, phosphorous, and calcium. The greater osmolality of MPS compared to CES or water likely contributed to the reduced urine production and greater fluid retention.

Indeed, our findings show that the cumulative urine output over the 4 h after ingestion of MPS was significantly lower than either water or CES (Figure 2A) and was accompanied by a longer time spent in positive fluid balance (Figure 2B). These results are consistent with prior studies showing that beverages with higher electrolyte concentrations and osmolality promote increased fluid retention in young adults [20]. The increased fluid retention with MPS resulted in an increased BHI across the entire 4 h time course compared to water and CES (Figure 3). Such an increase in fluid retention could be due to slower gastric emptying following ingestion of a high-osmotic solution, and we cannot rule out that possibility based on the data collected in this study [30].

However, it is unlikely that differences in gastric emptying played a major role in influencing the BHI because other investigators have reported that the primary factor affecting gastric emptying is the energy content of the beverage, even when the osmolalities of the test beverages varied widely. Consumption of 500 mL of isocaloric beverages displayed similar gastric emptying rates, despite large differences in beverage osmolalities [31], and impairments in gastric emptying were not seen at or below beverage glucose concentrations of 6% and osmolalities of 350 mosm/kg [32]. The energy content of the MPS tested here was 27% lower than that of the CES, suggesting that fluid from MPS did not remain in the stomach longer. Additional research is warranted to discern how these differences in BHI are influenced by differences in the rates of gastric emptying, absorption in the proximal small intestine, and renal urine production [33].

Serum osmolality was significantly elevated in the MPS trial compared to both CES and water at 60 min post-ingestion. This was driven, at least in part, by an increased osmolality of the MPS beverage. Intra-individual differences in serum osmolality between trials may explain some of the within-subject variability in urine output between beverages at earlier timepoints (i.e., 120 min), thus influencing BHI, although this variability appears to decrease at later timepoints (i.e., 240 min). Serum potassium concentration in the MPS trial was likewise elevated compared to water and CES in the first hour post-consumption. On the other hand, serum sodium concentration was only significantly elevated in the MPS trial compared to water and CES at 30 min, and was actually lowered compared to the CES trial at 60 min.

One important aspect of efficacious hydration is an expansion of extracellular fluid, specifically in the vascular compartment. There was an expansion of plasma volume (∆PV) in the MPS trial beginning at 30 min and continuing for the duration of the study, though this mildly increased plasma volume was not different from that of the water trial at any time point. Sustained elevations in PV, i.e., in 120- and 240-min responses are shown in Figure 5. The %∆PV was lower for MPS than CES over the initial 10–15 min after drinking but results were highly variable (data not shown), which may reflect a slower initial rate of gastric emptying in response to the higher osmolality of the MPS beverage [32]. Although subjects were in negative net fluid balance by the end of the study, indicating a greater excretion of fluid than what was consumed, there was a sustained plasma volume expansion of approximately 3–5% in the CES and MPS beverages at 240 min. The sustained mild PV expansion over at least 4 h after drinking MPS and CES likely indicates that the increased concentration of serum electrolytes helped retain some of the ingested fluid in the vascular space and possibly promoted some osmotic pull of water from the intracellular space into the vascular space [34], allowing for hemodilution, an important component of efficacious hydration [35].

The dairy-based MPS beverage in this study was approximately 4% glucose/galactose. In comparison, the carbohydrate–electrolyte solution (CES) tested in this study was 6% sucrose/glucose. It is thus important to elucidate potential differences in the glycemic load stemming from these differences in carbohydrate composition between beverages, which may be a particularly important consideration for populations who are at risk of metabolic dysfunction. The immediate rise in glucose concentration following MPS ingestion was blunted relative to the CES trial (Figure 5). Additionally, the plasma glucose concentrations returned to baseline sooner after MPS consumption compared to CES consumption, which showed an overshoot below baseline values at 60 min. These plasma glucose profiles may be attributable to the lowered total carbohydrate load and lower glucose concentration in the MPS beverage compared to the CES beverage, supporting its efficacy as a potential lower-glycemic alternative to traditional carbohydrate-based sports beverages.

Important considerations for the benefits of a hydration-promoting beverage include the taste, consistency, and thirst-quenching qualities of the beverage. As such, subjects in the current study completed a sensory evaluation survey for each beverage (data not displayed) regarding qualitative aspects, such as overall likability, taste, sweetness, aroma, and thirst-quenching properties. There were no statistical differences in responses among beverages.

### Limitations

In prior BHI studies [14,18], hydration solutions have been stored at approximately 4–6 °C. All beverages utilized in the current study were stored at 16–20 °C to prevent greater pressor responses to ingestion of cold beverages compared to room-temperature beverages [36], which may negatively impact effective venous blood sampling within the first 30 min post-ingestion. While the present study used a prescribed drinking protocol, it has previously been reported that stimulation of cold sensitive oropharyngeal receptors results in lowered ad libitum fluid consumption in humans, and that optimal water temperature to encourage ad libitum consumption is approximately 15 °C [37]. However, ad libitum consumption of either 4 or 20 °C water displayed no differential influence on hydration status in mildly dehydrated young adults [38]. The current recommendation of the American College of Sports Medicine is that ingested fluids for the purpose of hydration should be at ambient temperatures between 15 and 22 °C [39]. Therefore, we determined that storing beverages at room temperature was justified. Another limitation of this study, inherent to all BHI studies, is that the location of the fluid remaining in the body is unknown. BHI is determined by differences in the rates of gastric emptying, fluid absorption in the proximal small intestine, and renal urine production. In that regard, future studies measuring the gastric residual contents and duodenal constituent concentrations are warranted.

Although it was outside the scope of the current study to measure circulating concentrations of hormones associated with fluid balance maintenance, it is possible that circulating vasopressin influenced fluid retention in the current study. Following water consumption, there is a rapid decrease in plasma vasopressin concentrations independent of gastrointestinal absorption rate [40,41,42,43]. Additionally, these changes appear to occur prior to changes in plasma osmolality [44,45]. In a prior study examining the potential role of plasma vasopressin in plasma volume changes following consumption of either a glucose polymer–electrolyte solution or water, there were no differences in plasma vasopressin concentrations between the two trials despite a higher plasma volume in the glucose polymer–electrolyte trial compared to the water trial [46]. However, there were no changes in plasma osmolalities in either trial in that study, whereas in the current study there was an increase in plasma osmolality in the MPS trial. Future investigation is warranted to examine the potential role of vasopressin in mediating changes in plasma volume following consumption of beverages with a wide range in osmolalities.

Approximately 36% of the United States population is affected by lactose malabsorption to some degree [47], which may lead to gastrointestinal (GI) discomfort following dairy consumption. This is especially important in individuals who are exercising, as GI discomfort may impair performance. Thus, it is beneficial to explore potential dairy-based alternatives that can provide the same hydrating capacity of milk without the GI discomfort. The current MPS beverage consists of 2% glucose and 2% galactose and is protein- and fat-free. Milk typically contains 4–6% lactose. Although subjects in the current study did not report any GI discomfort at any point following consumption of the MPS beverage, no subjects in the study reported any history of intolerance to dairy-based products.

Subjects in this study started drinking in a euhydrated state and remained at rest for 4 h post-ingestion. When there is any suggestion that individuals may be even mildly dehydrated, clinicians often order prescribed drinking as a first step toward restoring or assuring euhydration. CES solutions are often the drink of choice in such conditions but cross-beverage comparisons have rarely been conducted. This adds situational validity to the BHI approach. However, it is unknown how these findings translate to maintenance of, or return to, euhydration under stressed conditions such as during and after exercise in the heat. Few investigations have examined the efficacy of either skim [48] or low-fat [49] milk for rehydration during or following acute bouts of exercise. Skim milk, both alone and with added sodium, resulted in a lower urine output compared to a traditional sports drink or water in the 4 h following cycling-induced dehydration [49], though this response was attributed to the high protein concentration of milk. The solution tested in our study contained milk permeate, an ultrafiltrate of milk which is both protein- and fat-free, with the approximate mineral content of milk, and equivalent sodium concentration of common sports drinks. Future investigation is warranted to examine the efficacy of this milk permeate solution as a both a beverage for consumption during and after exercise.

## 5. Conclusions

In summary, a novel dairy-based beverage containing milk permeate promoted better body fluid retention over 4 h post-ingestion. MPS had a lower cumulative urine output, maintained positive fluid balance longer, and thus had a higher beverage hydration index than that of a traditional carbohydrate-based electrolyte beverage or water. The increased fluid retention properties associated with ingestion of the milk permeate solution is likely attributable to its greater total mineral content and higher osmolality. In addition, the initial increase in plasma glucose concentration was lower following consumption of the milk permeate solution compared to the carbohydrate-based electrolyte solution. Together, these findings indicate that a dairy-based beverage containing milk permeate may serve as an efficacious alternative to traditional carbohydrate–electrolyte solutions in healthy young adults at rest. Future research is needed to investigate the efficacy of this milk permeate solution during and following physical activity or environmental stress, or in clinical populations.

## Figures and Tables

**Table 1:** Subject Characteristics.

	Mean	Range
**n (M/F)**	12 (6/6)	
**Age (years)**	23	20–26
**Weight (kg)**	69.6	52.9–94.6
**BMI (kg·m^−2^)**	23.7	20.5–29.9
**Systolic BP (mmHg)**	116	100–130
**Diastolic BP (mmHg)**	75	62–82
**HR (beats·min^−1^)**	65	56–72
**Total cholesterol (mg·dL^−1^)**	169	123–264
**HDL-C (mg·dL^−1^)**	56	41–74
**LDL-C (mg·dL^−1^)**	94	56–192
**HbA1C (%)**	5.0	4.8–5.5

Values displayed as mean and range and were obtained during the pre-study screening visit. BMI, body mass index; BP, blood pressure; HR, heart rate; HDL, high-density lipoproteins; LDL, low-density lipoproteins; HbA1c, glycated hemoglobin.

**Table 2:** Beverage Composition.

Beverage	CHO (%)	Energy (kcal/L)	Sodium, mmol/L	Potassium, mmol/L	Osmolality, mosm/kg
Water	0	0	0	0	0
CES	6	220	20	3.2	326 ± 3
MPS	4	160	21	28	621 ± 5

CES, carbohydrate–electrolyte solution; MPS, milk permeate solution. CHO, carbohydrate content. Values for CHO, kcal, sodium, and potassium are label values. Osmolality was measured in triplicate in our laboratory using a freezing-point osmometer (Model 3320, Advanced Instruments, Inc., Norwood, MA, USA).

**Table 3:** Baseline Characteristics by Trial.

	Water	CES	MPS
	Mean ± SD	Mean ± SD	Mean ± SD
**Fasting glucose (mg·dL^−1^)**	87 ± 6	88 ± 5	88 ± 6
**Creatinine (mg·dL^−1^)**	0.86 ± 0.17	0.87 ± 0.18	0.89 ± 0.21
**Serum Na^+^ (mmol·L^−1^)**	138 ± 2	137 ± 2	138 ± 2
**Urine Na^+^ (mmol·L^−1^)**	84 ± 48	91 ± 79	75 ± 49
**S_osm_ (mOsm·kg^−1^)**	291 ± 5	291 ± 5	291 ± 5
**U_osm_ (mOsm·kg^−1^)**	511 ± 296	566 ± 301	574 ± 342
**Urine Specific Gravity**	1.015 ± 0.008	1.017 ± 0.008	1.017 ± 0.009

eGFR, estimated glomerular filtration rate; Na^+^, sodium; S_osm_, serum osmolality; U_osm_, urine osmolality. Serum and urine values were calculated from the “pre” time point urine samples of all trials. There were no significant differences in any baseline measurements among trials.

**Table 4:** Serum and urine electrolyte, osmolality, and urine free water clearance (CH_2_O) values. Values are means ± SD. Differences among beverages were assessed by two-way ANOVA. * *p* < 0.05 compared to water; † *p* < 0.05 compared to CES; ‡ *p* < 0.05 compared to Pre.

			Pre	0 min	30 min	60 min	120 min	180 min	240 min
Serum									
**Urine**	**Sodium (mmol/L)**	Water	138 ± 2	136 ± 3 ‡	135 ± 2 ‡	136 ± 2	137 ± 2	138 ± 2	138 ± 3
		CES	137 ± 2	136 ± 1 ‡	137 ± 2 *	138 ± 1 *	138 ± 1	138 ± 2	138 ± 1
		MPS	138 ± 2	135 ± 5 ‡	137 ± 2 *	136 ± 4 †‡	137 ± 1	138 ± 2	138 ± 2
	**Potassium (mmol/L)**	Water	4.1 ± 0.1	4.3 ± 0.2 ‡	4.2 ± 0.3	4.3 ± 0.3 ‡	4.2 ± 0.4 ‡	4.1 ± 0.4	4.1 ± 0.3
		CES	4.0 ± 0.2	4.0 ± 0.3 *	3.9 ± 0.2 *	4.1 ± 0.2 *	4.3 ± 0.2 ‡	4.4 ± 0.3 *‡	4.3 ± 0.3 *‡
		MPS	4.0 ± 0.3	4.3 ± 0.4 †‡	4.4 ± 0.4 *†‡	4.4 ± 0.4 *†‡	4.5 ± 0.3 *†‡	4.4 ± 0.2 *‡	4.4 ± 0.2 *‡
	**Osmolality (mosm/kg)**	Water	291 ± 5	288 ± 6 ‡	287 ± 6 ‡	287 ± 9 ‡	291 ± 6	291 ± 5	292 ± 6
		CES	291 ± 5	292 ± 4 *	290 ± 5 *	290 ± 4 *	292 ± 3	292 ± 5	293 ± 5
		MPS	291 ± 5	293 ± 6 *‡	293 ± 7 *	294 ± 6 *†‡	293 ± 6	294 ± 3 ‡	293 ± 3 ‡
	**Sodium (mmol/L)**	Water	72 ± 49	42 ± 36		28 ± 47	24 ± 19	62 ± 20	95 ± 42
		CES	87 ± 73	38 ± 30 ‡		29 ± 37 ‡	26 ± 17 ‡	59 ± 30 ‡	103 ± 28
		MPS	71 ± 50	61 ± 47 ‡		46 ± 32 ‡	78 ± 42 *†‡	100 ± 36 *†‡	115 ± 40 ‡
	**Potassium (mmol/L)**	Water	28.5 ± 23.2	29.0 ± 18.2		10.2 ± 2.8	11.9 ± 6.8	29.6 ± 22.7	42.6 ± 33.5
		CES	33.3 ± 20.8	21.4 ± 21.7		3.1 ± 1.5 ‡	10.2 ± 4.9 ‡	40.5 ± 46.7	55.3 ± 32.5 ‡
		MPS	28.5 ± 20.7	32.3 ± 33.5		14.5 ± 13.5 ‡	35.5 ± 27.0 *†‡	51.3 ± 29.8 *‡	63.5 ± 33.3 *‡
	**Osmolality (mosm/kg)**	Water	511 ± 282	308 ± 265 ‡		144 ± 217 ‡	113 ± 23 ‡	305 ± 305 ‡	465 ± 465
		CES	564 ± 288	352 ± 298 ‡		71 ± 18 ‡	130 ± 45 ‡	309 ± 68 ‡	540 ± 115
		MPS	563 ± 345	387 ± 305 ‡		226 ± 147 †‡	370 ± 166 *†‡	537 ± 138 *†	635 ± 134 *
	**CH_2_O**	Water		3 ± 5		6 ± 2	4 ± 1	1 ± 2	−1 ± 1
	**(mL/min)**	CES		2 ± 4		7 ± 2	4 ± 2	0 ± 1	−1 ± 0
		MPS		1 ± 5 *†		2 ± 3 *†	0 ± 3 *†	−2 ± 1 *	−2 ± 1
